# Advances in Preventive Therapy for Estrogen-Receptor-Negative Breast Cancer

**DOI:** 10.1007/s12609-014-0144-1

**Published:** 2014-03-25

**Authors:** Beate C. Litzenburger, Powel H. Brown

**Affiliations:** Department of Clinical Cancer Prevention, The University of Texas MD Anderson Cancer Center, 1155 Herman P. Pressler, Unit 1360, Houston, TX 77030 USA

**Keywords:** Breast cancer, Prevention, Estrogen receptor-negative breast cancer, Triple-negative breast cancer

## Abstract

Preventing breast cancer is an effective strategy for reducing breast cancer deaths. The purpose of chemoprevention (also termed preventive therapy) is to reduce cancer incidence by use of natural, synthetic, or biological agents. The efficacy of tamoxifen, raloxifene, and exemestane as preventive therapy against estrogen-receptor (ER)-positive breast cancer is well established for women at increased risk for breast cancer. However, because breast cancer is a heterogeneous disease, distinct preventive approaches may be required for effective prevention of each subtype. Current research is, therefore, focused on identifying alternative mechanisms by which biologically active compounds can reduce the risk of all breast cancer subtypes including ER-negative breast cancer. Promising agents are currently being developed for prevention of HER2-positive and triple-negative breast cancer (TNBC) and include inhibitors of the ErbB family receptors, COX-2 inhibitors, metformin, retinoids, statins, poly(ADP-ribose) polymerase inhibitors, and natural compounds. This review focuses on recent progress in research to develop more effective preventive agents, in particular for prevention of ER-negative breast cancer.

## Introduction

Given the global increase in cancer incidence with its associated morbidity, mortality, and enormous treatment costs, there is increasing interest in strategies for disease prevention. Breast cancer is the most common form of cancer among women worldwide, with an estimated 232,340 new cases among US women in 2013 alone [1]. In the US the incidence has been stable over the last decade, although increasing almost everywhere throughout the world. Although breast cancer mortality is declining [1, 2], preventing breast cancer is the most effective way of reducing breast cancer death. Primary prevention focuses on preventing cancer from developing or delaying the development of a malignancy. Prevention strategies encompass avoidance of known carcinogens (e.g. benzene), promotion of behavioral strategies to reduce risk through diet, exercise, limited alcohol consumption, and no tobacco use. For individuals with a particularly high risk of breast cancer, management includes genetic screening, early detection by use of mammography and breast MRI, use of preventive medications, and such surgical strategies as bilateral mastectomy.

Prevention of either the initial phases of carcinogenesis or the progression of premalignant cells to invasive disease, thereby reducing the risk of cancer, can be achieved by pharmacological means, commonly referred to as chemoprevention (also termed preventive therapy). The purpose of chemoprevention is to reduce cancer incidence by use of natural, synthetic, or biological agents. The value of this approach has been demonstrated in breast cancer prevention trials which have primarily focused on endocrine intervention by use of selective estrogen receptor modulators (SERMs; for example tamoxifen) and aromatase inhibitors (AIs; for example exemestane) (comprehensively reviewed elsewhere [3]).

**Fig. 1:**
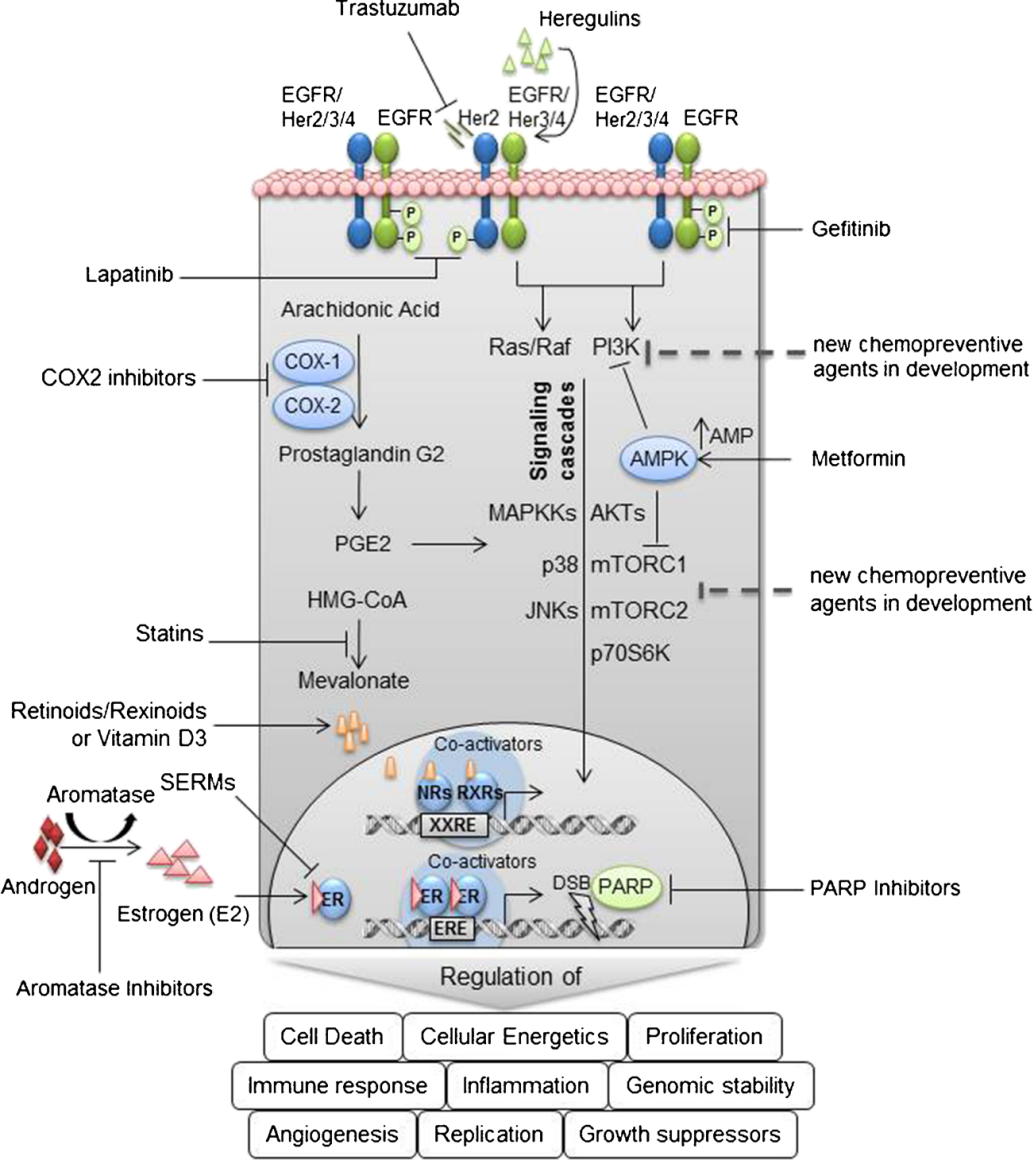
Molecular targets for breast cancer chemoprevention. *Solid lines* indicate drugs currently used in preclinical or clinical chemopreventive studies whereas *dotted lines* indicate promising drugs for future chemopreventive studies. *mTORC1*, mammalian target of rapamycin complex 1 composed of mTOR, Raptor, MLST8, PRAS40, and DEPTOR [121]; *mTORC2*, mammalian target of rapamycin complex 2 composed of mTOR, RICTOR, mLST8 and mSIN1 [122].

Because breast cancer includes both estrogen-receptor-positive (ER-positive) and estrogen-receptor-negative (ER-negative) subtypes, distinct chemopreventive approaches may be required for effective prevention of each subtype. Promising approaches to prevention of ER-negative breast cancer include targeting molecules critical for the growth and progression of ER-negative tumors, for example inhibitors of the ErbB family of receptors, cyclooxygenase-2 (COX-2) inhibitors, metformin, retinoids, statins, poly(ADP-ribose) polymerase, natural compounds, and others (Fig. 1).

Studies leading to successful FDA approval of anti-estrogenic drugs for prevention of ER-positive breast cancer among high-risk women are reviewed briefly below. However, these drugs do not reduce the risk of ER-negative breast cancer, which accounts for 20–30 % of breast cancers. Therefore, we also review chemopreventive approaches to reducing the risk of ER-negative breast cancer.

### Subtypes of Breast Cancer

The classical breast cancer subtypes are based on assessment of clinical and pathological factors, for example ER, progesterone receptor (PR), or HER2 status, tumor grade, tumor size, and the presence or absence of lymph node metastasis. However, microarray-based gene-expression studies have led to the identification of molecular subtypes (basal-like, HER2-enriched, luminal, and normal breast-like) with distinctly different survival and treatment response [4, 5]. Luminal tumors are ER and PR-positive whereas HER2-positive tumors arise from overexpression or amplification of the EGFR family of receptor tyrosine kinases, particularly HER2 (also called ErbB2, neu). HER2 overexpression is present in approximately 20–30 % of all human breast tumors, particularly those that are ER-negative [6]. Triple-negative breast cancer (TNBC) is characterized by lack of expression of ER, PR, and HER2 and accounts for approximately 15–20 % of all breast cancer diagnoses. TNBC is molecularly a heterogeneous disease. Most basal-like breast cancers (∼80 %) are of the triple-negative phenotype. More recently, TNBC has been divided into six distinct subtypes:

1. immunomodulatory;
2. mesenchymal;
3. mesenchymal stem-like;
4. luminal androgen receptor;
5. basal-like 1; and
6. basal-like 2 [7].

Therefore, the development of effective preventive agents suppressing the development of TNBC remains a challenge, because of the heterogeneity of the disease.

### Prevention of ER-Positive Breast Cancers

ER and its ligand estrogen are key regulators in breast cancer carcinogenesis, and modulation of the receptor or reduction of estrogen are strategies for reduction of breast cancer risk. Clinical studies testing SERMs, for example tamoxifen, or AIs, for example anastrozole, for treatment of early breast cancer laid the foundation for breast cancer prevention in the future.

The National Surgical Adjuvant Breast and Bowel Project (NSABP) BCPT (also known as P-1) trial demonstrated that tamoxifen reduced the risk of invasive breast cancer by 49 % versus placebo among women at risk [8]. The STAR trial demonstrated that raloxifene was as effective as tamoxifen, and reduced breast cancer risk in postmenopausal women by approximately 50 % [9]. The results of the P-1 and STAR trials led to FDA approval of tamoxifen or raloxifene for use for breast cancer prevention among high-risk women. More recently, the 81-month follow-up study demonstrated that after stopping drug treatments after five years the cancer-preventive effect of tamoxifen persisted whereas the cancer-preventive effect of raloxifene diminished over time. Raloxifene retained only 76 % of the effectiveness of tamoxifen at prevention of invasive breast cancer [10]. However, raloxifene was generally less toxic than tamoxifen, which is of particular interest for high-risk postmenopausal women with intact uterus who are concerned about the risk of hot flushes, thromboembolic side effects, and endometrial cancer, whereas tamoxifen may be preferred for a high-risk postmenopausal woman without a uterus, and for premenopausal women [11].

Clinical trials of AIs as adjuvant therapy demonstrated their cancer-preventive potential, because they effectively prevent breast cancer recurrence [12, 13] and the development of second primary contralateral tumors [12]. The NCIC-MAP.3 trial tested the aromatase inhibitor exemestane versus placebo among postmenopausal high risk women for up to five years of treatment [14]. In this trial, exemestane reduced the incidence of invasive breast cancer by 65 % and the incidence of ER-positive invasive breast cancer by 73 %. Adverse events, for example endometrial cancers and thromboembolic events, which are usually associated with tamoxifen treatment, were not reported. On the other hand, exemestane is associated with hot flushes and bone pain, and increases the risk of bone fracture [14]. The IBIS-II trial tested the cancer-preventive effect of another AI, anastrozole, among postmenopausal women at risk for breast cancer. The results of this trial, in which women were treated with 1 mg oral anastrozole or placebo every day for five years, were recently reported [15]. Anastrozole reduced the incidence of breast cancer by 53 % among high-risk postmenopausal women [15].

Although AIs are not yet FDA-approved for breast cancer prevention, these drugs are already being used off-label for this purpose. The success of SERMS and AIs demonstrates that preventive therapy for breast cancer is possible.

### Prevention of HER2-Positive Breast Cancer

Several different ErbB family receptor inhibitors are FDA-approved for clinical use. The monoclonal antibodies cetuximab, trastuzumab, and pertuzumab are directed against the extracellular domain of their target receptor proteins and prevent receptor interaction with growth factor and/or dimerization with other receptors whereas the small-molecule inhibitors lapatinib, gefitinib, and erlotinib interfere with the kinase activity of their target proteins (Fig. 1) [16]. Given the activity of these drugs in cancer treatment research is now focused on investigating in particular oral HER2 and EGFR receptor tyrosine kinase inhibitors in preclinical and early clinical trials as breast cancer-preventive drugs.

In preclinical mouse models our group has shown that treatment with gefitinib, an EGFR kinase inhibitor, delayed the development of spontaneous ER-negative, HER2-positive mammary tumors in the MMTV-ErbB2 transgenic mouse model [17] (median time to tumor formation in the control group 230 days versus 310 days in the high-dose gefitinib group, *p* < 0.001) (Table 1). Furthermore, gefitinib reduces proliferation and tumor multiplicity (Table 1) [18, 19]. Likewise, Lapatinib, a dual EGFR and ErbB2 receptor tyrosine kinases inhibitor, or vehicle was administered long-term to MMTV-ErbB2 transgenic mice before the development of tumors. Lapatinib inhibited the formation of premalignant lesions in the mammary gland and reduced ER-negative and HER2-positive tumor development by 69 % (Table 1) [20].

**Table 1:** Selected promising agents for prevention of ER-negative breast cancer in preclinical studies

Drug	Model	Study design	Results	*P*-value	Ref.
Anti EGFR/HER2 inhibitors					
Gefitinib	MMTV-Erb2 mice	Gefitinib (10 mg kg^−1^ or 100 mg kg^−1^) versus vehicle Treatment time: 9 months (3–12 months of age)	Delay in MTTF: 100 mg kg^−1^ gefitinib: 310 days Vehicle: 230 days	*P* < 0.001	[17]
Gefitinib	NeuT transgenic mice	Gefitinib (75 mg kg^−1^ to 135 mg kg^−1^) versus vehicle Treatment time: 8–9 weeks	Reduced tumor multiplicity by: 83 %	*P* < 0.0001	[18]
Gefitinib	DCIS transplanted in BALB/c^nu/nu^ mice	Gefitinib (10 to 200 mg kg^−1^) versus vehicle Treatment time: 14–28 days	Reduction in proliferation by: 56 % in EGFR-positive DCIS	*P* ≤ 0.001	[19]
Lapatinib	MMTV-Erb2 mice	Lapatinib (30 mg kg^−1^ or 75 mg kg^−1^) versus vehicle Treatment time: 12 months (3–15 months of age)	Reduction in tumor incidence by: 69 % (75 mg kg^−1^ lapatinib versus vehicle at 418 days of age)	*P* < 0.001	[20]
COX-2 Inhibitor					
Celecoxib and bexarotene	MMTV-Erb2 mice	Celecoxib (500 ppm), Bexarotene (10 mg kg^−1^) alone or in combination versus vehicle Treatment time: 8 to 98 weeks of age	Delay in MTTF: Celecoxib and Bexarotene: >600 Bexarotene: 420 Celecoxib: 285 Vehicle: 304	*P* < 0.0001 *P* = 0.0085 *P* = 0.26	[32]
Metformin					
Metformin and melatonin	MMTV-Erb2 mice	Metformin (100 mg kg^−1^), Melatonin (2 mg/l) or the combination versus vehicle Treatment time: 2 months of age to death	Delay in tumor latency by: Metformin: 13 %	*P* < 0.05	[45]
Rexinoid					
Bexarotene	p53 null mammary gland mouse model	Bexarotene (10 mg kg^−1^ or 100 mg kg^−1^) versus vehicle Treatment time: 3 months of age until termination of the experiment	Reduction in tumor incidence (at 60 weeks of age) by: 75 % (100 mg kg^−1^ bexarotene versus vehicle)	*P* < 0.05	[71]
UAB30	MNU-treated Sprague–Dawley rats	Methyl-UAB30 (200 mg kg^−1^), Bexarotene (150 or 15 mg kg^−1^), 9-*cis*-RA (60 mg kg^−1^) versus vehicle Treatment time: 126 days	Reduced tumor multiplicity by: 9-*cis*-RA: 51 % Bexarotene 15 mg kg^−1^: 38 % Bexarotene 150 mg kg^−1^: 70 % 4-methyl-UAB30: 74 %	N/A	[76]
LG100268	MMTV-Erb2 mice	LG100268 (10 mg kg^−1^ or 100 mg kg^−1^) versus vehicle Treatment time: 17 months (3–20 months of age)	Delay in MTTF: 100 mg kg^−1^ LG100268: 430 days 10 mg kg^−1^ LG100268: 357 days Vehicle: 217 days	*P* < 0.0001	[78]
LG100268 and Tamoxifen	p53 null mammary gland mouse model	Tamoxifen (2.5 mg), LG100268 (50 mg kg^−1^) or in combination versus vehicle Treatment time: 18 weeks (11 – 29 weeks of age)	Tumor development (at 60 weeks of age): Vehicle: 52 % Tamoxifen: 42 % LG100268: 37 % Combination: 13 %	*P* = 0.014	[79]
PARP inhibitors					
Olaparib and Veliparib	Brca1^−/−^ MMTV-Cre p53^+/−^ mice	Olaparib (200 mg kg^−1^) or veliparib (100 mg kg^−1^) versus vehicle Treatment time: 10 weeks of age to death	Delay in tumor development: Olaparib: 6.6 weeks Veliparib: 2.4 weeks	*P* < 0.005	[96]
mTOR inhibitors					
Rapamycin	MMTV-Neu^ndl^-YD5 mice MMTV-VEGF-164	Rapamycin (0.75 mg kg^−1^) versus vehicle Treatment time: max. 94 days (38 up to 132 days postpartum	Reduction in tumor mass by: 75 %	*P* < 0.0007	[101]
Rapamycin	Injection of Met-1 cells in FVB/N or Cav-1^−/−^ mice	Rapamycin (2.78 μg kg^−1^) versus vehicle Treatment time: 5 weeks	4.7-fold reduction in tumor growth in Cav-1^−/−^ mice	*P* < 0.001	[102]
Triterpenoid					
CDDO-Me	MMTV-Erb2 mice	CDDO-Me (60 mg kg^−1^), LG100268 (20 mg kg^−1^), or the combination versus vehicle Treatment time: 45 weeks (10 – 55 weeks of age)	Prevention of tumor development: CDDO-Me: 50 % LG100268: 57 % Combination: 87.5 %	*P* < 0.05	[70]

Abbreviations: MTTF, median time to tumor formation; DCIS, ductal carcinoma in situ; EGFR, epidermal growth factor receptor; MNU, methylnitrosourea; N/A, not available; PARP, poly ADP ribose polymerase; CDDO-Me, 2-cyano-3,12-dioxooleana-1,9(11)-dien-28-oic acid methyl ester

Lapatinib was further investigated in clinical trials. In a phase II trial lapatinib (1,500 mg once a day) or placebo was investigated for treatment of 60 women with HER2-positive ductal carcinoma in situ (DCIS) for three weeks before surgical resection [21]. Primary endpoint results revealed reduced proliferation of breast epithelial cells, adjacent ductal intraepithelial neoplasia, and distant ductal hyperplasia (indicated by immunohistochemical staining for KI67) (Table 2) [21]. Another similar trial among women with HER2 or EGFR-positive DCIS is currently testing the effect of lapatinib (1000 mg day^−1^) versus placebo for two to six weeks before surgical excision. The primary objective is to determine the rate of proliferation of DCIS breast cancer cells measured by immunohistochemical staining for Ki67. Secondary endpoints include investigation of whether lapatinib affects the incidence of DCIS observed at time of surgical excision (Table 2).

**Table 2:** Selected clinical studies investigating promising agents for the prevention of ER-negative breast cancer

Drug	Population	*N*	Study design	Primary endpoint	Results	HR(95%CI)/*P*-value	Ref.
Lapatinib							
Completed	HER2-positive breast cancer	60	Phase IIb Lapatinib (1,500 mg/d) versus placebo Treatment time: 3 weeks followed by surgery	Proliferation (KI67)	Biomarker reduction (Ki67) in: breast cancer tissue adjacent DIN distant DH without atypia	*P* = 0.008 *P* = 0.067 *P* = 0.006	[21]
LAPIS trial NCT00555152	HER2- or EGFR-positive DCIS	120	Phase II Lapatinib (1000 mg/d) versus placebo Treatment time: 2–6 weeks followed by surgery	Proliferation (KI67)	Not yet available	NA	
Trastuzumab							
Completed	HER2-positive DCIS	24	Phase II Trastuzumab single dose therapy (8 mg kg^−1^) versus placebo Treatment time: 14–28 days followed by surgery	Proliferation (KI67)	Biomarker change: No changes in proliferation, apoptosis or pathologic response Increased antibody-dependent cell mediated cytotoxicity	*P* > 0.5 *P* < .01	[22]
NSABP43 NCT00769379	HER2-positive DCIS	2000	Phase III Trastuzumab (2 doses at wk1 and wk4) and radiation therapy versus radiation therapy alone Treatment time: 5–6 weeks	Time to IIBCR-SCR-DCIS	Not yet available	NA	
Celecoxib							
Completed	Early stage breast cancer	37	Phase II Celecoxib (400 mg bid) versus placebo Treatment time : 2–3 weeks followed by surgery	Proliferation (KI67)	Biomarker change (Ki67) relative to baseline: Significant change in Ki67 between groups	*P* = 0.029	[33]
NCT00291694	Hyperplasia of the breast	72	Phase II Celecoxib (400 mg bid) versus placebo Treatment time: 12 months	Proliferation (KI67)	Not yet available	NA	
NCT00056082	Premenopausal, at risk for ER-negative breast cancer	110	Phase II Single arm celecoxib (400 mg bid) Treatment time: 12 months	Proliferation (KI67)	Not yet available	NA	
REACT trial ISRCTN48254013	Primary breast cancer	2590	Phase III Celecoxib (400 mg bid) versus placebo (2:1 ratio) Treatment time: 2 years	DFS	Not yet available	NA	
Metformin							
Completed	Non-diabetic with operable invasive breast cancer	200	Pre-operative, window of opportunity study: Metformin (850 mg o.d. days 1–3, and 850 mg bid days 4–28) versus placebo Treatment time: 28 days	Proliferation (KI67)	Biomarker change (Ki67): Overall: no significant KI67 change	95 % CI, −5.6 % to 14.4 %	[47]
Completed	Non-diabetic with operable invasive breast cancer	55	Pre-operative, window of opportunity study: Metformin (500 mg o.d. for wk1, 1 g bid. for wk2) versus placebo	Proliferation (KI67)	Biomarker reduction (Ki67): Metformin: 3.4 %	*P* = 0.027	[48]
NCIC MA.32 NCT01101438	Non-diabetic subjects with Early-stage breast cancer	3582	Phase III Metformin (850 mg bid, 1 month ramp up in dose) vs. placebo Treatment time. 5 years	IDFS	Not yet available	NA	[51]
Fenretinide							
Completed	Stage-I breast cancer or DCIS without adjuvant therapy	1739	Phase III Fenretinide versus no treatment Treatment time: 5 years	Reduction in contralateral or ipsilateral breast cancer	Prevention of incidence: All breast cancers (contralateral and ipsilateral): 17 % Contralateral breast cancer: 10 % Ipsilateral breast cancer: 23 %	HR = 0.83, 0.67–1.03). HR = 0.90 (0.65–1.26) HR = 0.77 (0.58–1.02)	[69]
Statin (fluvastatin)							
Completed	DCIS or stage I breast cancer	45	Pre-operative, window of opportunity study: Fluvastatin (80 mg day^−1^ or 20 mg day^−1^) Treatment time: 3–6 weeks followed by surgery	Proliferation (KI67)	Biomarker reduction (Ki67) high grade tumors: 7.2 % Biomarker increase (apoptosis) high grade tumors: 60 %	*P* = 0.008 *P* = 0.015	[93]
Selected natural compounds							
EGCG Completed	Stage I to III hormone receptor-negative breast cancer	1000	Phase IB dose escalation trial polyphenon E ( 400–800 bid) versus placebo Treatment time: 6 months	MTD	MTD: 600 mg bid	NA	[107]
Vitamin D3 VITAL trial NCT01169259	Healthy women and men	20000	Phase III VitD3 (2000 IU), *n* − 3 fatty acids (1000 mg), combination vs. placebo Treatment time: 5 year	Cancer incidence and cardiovascular disease	Not yet available	NA	

Abbreviations: CI, confidence interval; HR, hazard ratio; DCIS, ductal carcinoma in situ; DIN, ductal intraepithelial neoplasia; DH, ductal hyperplasia; Wk, week; NSABP43, National Surgical Breast and Bowel Project 43; IIBCR-SCR-DCIS, second breast cancer recurrence, skin cancer recurrence, or ipsilateral ductal carcinoma in situ; DFS, disease-free survival; IDFS, invasive disease-free survival, MTD, maximum tolerated dose; 25(OH)D, 25-hydroxyvitamin D

Treatment of HER2-positive DCIS with trastuzumab, a monoclonal antibody against HER2, was tested in a phase II trial in a neoadjuvant setting. Preoperative single-dose monotherapy with trastuzumab resulted in an immunologic response (increased antibody-dependent cell-mediated cytotoxicity) with no association in histologic, antiproliferative, or apoptotic changes (Table 2) [22]. In an ongoing phase III NSABP-43 trial the effect of trastuzumab is being assessed in women with HER2-positive DCIS after excisional surgery given concurrently with radiation or radiation alone. The primary objective is to determine whether trastuzumab prevents subsequent recurrence of ipsilateral breast cancer, ipsilateral skin cancer, or ipsilateral DCIS. One of the secondary objectives is to determine whether trastuzumab has a preventive effect in prolonging invasive or DCIS disease-free survival (Table 2).

In addition to FDA-approved HER2/EGFR inhibitors, such alternative approaches as use of Her-2/neu vaccines to manipulate the immune system with long-lasting anti-tumor effects are under investigation. Several systems have been developed to deliver tumor-associated antigens into the body, for example whole tumor cell vaccines, dendritic cell vaccines, viral vector vaccines, and peptide vaccines, the last being the focus of clinical investigations among high-risk women for prevention of HER2-positive breast cancer. The most studied HER2-derived peptide in clinical trials is E75 (NeuVax, HER2/neu 369–377), an immunogenic HLA class-I peptide that stimulates cytotoxic T lymphocytes. Results from phase II studies suggest that E75, in particular when administered in the adjuvant setting, prevents disease recurrence among selected high-risk patients [23, 24]. The first phase III clinical trial, called the PRESENT trial, will determine the efficacy and safety of E75 vaccine and evaluate and compare disease-free survival (DFS) among E75 vaccinated patients and control patients. Another ongoing phase II study will investigate the combination of immunotherapy with E75 and trastuzumab to prevent recurrence among high-risk HER2-positive breast cancer patients who are disease-free after standard of care therapy.

## Preventive Agents for ER-Negative Breast Cancer Including TNBC

### NSAIDs, Aspirin, and COX-2 Inhibitors

Increasing epidemiological, experimental, and clinical studies have demonstrated that nonsteroidal anti-inflammatory drugs (NSAIDs) have a preventive effect on the development of some malignancies including breast cancer. NSAIDs inhibit cyclooxygenase (COX), for which two isoforms, COX-1 and COX-2, have been described. COX-1 is constitutively produced in most cells whereas COX-2 is induced by mitotic signals and pro-inflammatory stimuli [25]. NSAIDs impair the transformation of arachidonic acid to prostaglandins (Fig. 1), prostacyclin, and thromboxanes. COX-2 is overexpressed in invasive breast cancer, and in DCIS and adjacent tissue, suggesting COX-2 is an important driver of mammary tumorigenesis [26].

Most traditional NSAIDs, for example aspirin, are nonselective inhibitors of both COX-1 and COX-2. Randomized studies have identified reduced cancer risk (e.g., lung, colon, breast) associated with long-term use of aspirin [27]. A meta-analysis showed that long-term aspirin intake is associated with a 10 % reduction in risk of breast cancer [28]. Extended follow-up revealed overall cancer mortality was reduced by approximately 20 % among people with regular intake of aspirin, with the greatest benefit seen in deaths from adenocarcinoma (36 % reduction) [27].

The selective COX-2 inhibitors celecoxib (Celebrex), rofecoxib (Vioxx), and valdecoxib (Bextra) have received approval from the FDA in the US for treatment of pain; rofecoxib and valdecoxib were, however, withdrawn worldwide by the manufacturer after an increased number of cardiovascular events was seen in placebo-controlled trials. Therefore, subsequent research has focused on investigating the cancer-preventive effectiveness of celecoxib in preclinical and clinical studies.

In preclinical studies treatment with celecoxib reduced mammary tumor incidence among treated mice [29, 30]. However, one study using the same mouse model showed that celecoxib was unable to prevent tumor development [31]. Our group showed celecoxib alone was ineffective but coadministration of celecoxib and bexarotene, a rexinoid, substantially delayed tumor development (Table 1) [32].

Recently, a phase II biomarker trial has demonstrated that celecoxib reduced proliferation in primary breast cancer tissues [33]. The first phase III clinical trial, called REACT, assessing the disease-free survival benefit of two years of celecoxib treatment among women with resected primary breast cancer is currently in progress (Table 2).

Although COX inhibitors have a significant chemopreventive effect on breast cancer risk, further clinical trials are needed. Moreover, concerns about rare serious toxicity, cardiovascular toxicity associated with COX-2 inhibitors, and other known potential side effects of treatment with NSAIDS, for example gastrointestinal bleeding and perforation, should be taken into account before routine implementation of NSAIDS as chemoprevention for breast cancer. To advance this field, new less toxic drugs targeting the COX2 pathway will need to be developed.

### Metformin

Metformin (1,1-dimethylbiguanide) is the most widely used first-line therapy of choice for type 2 diabetes mellitus. The primary target of metformin is AMPK in the mitochondria [34, 35]. Upon disruption of mitochondrial complex I, AMP/ATP and ADP/ATP ratios are increased which activates AMPK [34, 35] (Fig. 1). AMPK activation controls many metabolic processes, including fatty acid synthesis, gluconeogenesis in the liver, and glucose uptake in muscle (reviewed elsewhere [36]). However, the molecular mechanism of the preventive effect of metformin is still not understood. It is suggested that reduced insulin levels on treatment with metformin result in reduction of cell growth, thereby reducing tumorigenesis [37].

Epidemiological studies recently confirmed an association between type 2 diabetes and breast cancer risk, predominantly among postmenopausal women [38]. Moreover, other retrospective studies have demonstrated that metformin, in particular, reduces the risk of breast cancer compared with other antidiabetic therapy (e.g., insulin, alpha-glucosidase inhibitors, prandial glucose regulators, sulfonylureas, thiazolidinediones) [39–44]. In laboratory studies, metformin inhibits mammary tumor growth (Table 1) [45] and selectively targets tumor-initiating cells in this mouse model [46].

Because of these promising epidemiologic and preclinical data, several phase I and II trials were conducted to investigate its breast cancer-preventive effects [47–49]. Most of these studies were neoadjuvant “window of opportunity” studies among women with operable breast cancer and investigated a variety of biomarker changes after metformin administration (Table 2). Metformin reduced proliferation (KI67) and increased apoptosis (TUNEL staining) in invasive tumor tissue [49, 50]. Phase II and III clinical trials are currently in progress to further elucidate the cancer-preventive effect of metformin [51–55]. One important currently ongoing phase III study is the NCIC-MA.32 trial testing five years of metformin or placebo among women with early-stage breast cancer [51]. The primary outcome is invasive disease-free survival, with overall survival and contralateral breast cancer incidence as secondary endpoints (Table 2).

The results of these clinical trials will determine whether metformin will be useful for the prevention of breast cancer.

### Retinoids

Evidence from in-vitro studies suggest that retinoids control various signaling mechanisms irrespective of the ER/PR status of the cell, which makes them particularly promising drugs for prevention of ER-negative breast cancer. Retinoids are vitamin A analogues that regulate gene expression by binding to nuclear hormone receptors, including retinoic acid receptors (RARs) and retinoid X receptors (RXRs) [56]. Retinoids, for example all-*trans*-retinoic acid (ATRA), alitretinoin (9-*cis*-RA), and isotretinoin (13-*cis*-RA), can activate RAR and RXR, which heterodimerize and bind to the DNA on RA response elements to induce activation of genes involved in cellular proliferation, differentiation, and apoptosis [56].

Retinoids have been successfully used for prevention and treatment of cancers [57] and a variety of preclinical studies using mouse and rat models have demonstrated reduced mammary tumorigenesis because of the cancer-preventive effect of retinoids [58–60]. Preclinical studies also elucidated specific retinoid-mediated mechanisms of the cancer-preventive effect; these include:

- down-regulation of expression of COX2 and cyclin D1 [61];
- inhibition of AP1 transcription factor activity [62, 63];
- induction of cell cycle arrest at G1 [64, 65]; and
- overexpression of IGF binding proteins (IGF-BPs) 3 and 6 [63], RAR-beta [63] and TGF-beta [66].

One of the first chemoprevention trials among humans showed that 13-*cis*-RA prevented second primary tumors among head and neck patients [67]; for chemoprevention, however, the toxicity associated with 13-*cis*-RA treatment is not acceptable. Similarly, toxicity associated with 9-*cis*-RA treatment prevented further development of this agent as a standard chemopreventive drug [60, 67].

The synthetic retinoic acid, fenretinide, has been widely studied in clinical trials of chemoprevention, because of its favorable toxicity profile compared with the previously mentioned retinoids. In a phase III trial women with breast cancer were randomly assigned to receive no treatment or oral fenretinide (200 mg) daily for five years [68]. After a median follow-up of eight years, fenretinide treatment did not reduce the incidence of second breast cancers overall; when given to premenopausal women, however, fenretinide resulted in significant (38 %) reduction of the risk of second breast cancers (Table 2) [68, 69].

### Rexinoids

Synthetic “rexinoids” (e.g. bexarotene and LG100268) activate RXR without activating RAR nuclear receptors [70–73]. Bexarotene is FDA-approved for the treatment of cutaneous T-cell lymphoma; it has been tested for treatment of advanced breast cancer but was not effective as a single agent [74]. Our laboratory has previously shown that bexarotene can prevent ER-negative/HER2 positive mammary tumors in preclinical mouse models (Table 1) [73]. A breast cancer prevention trial of bexarotene among women at genetic risk showed that it reduced proliferation markers (Ki67, Cyclin D1) in breast tissues [75]. However, as with 13-*cis*-RA and 9-*cis*-RA, bexarotene had significant side effects, for example hypertriglyceridemia, which was reversible upon stopping the drug [75].

9cUAB30 is a synthetic analog of 9-*cis*-RA with little or no RAR-binding activity compared with 9-*cis*- RA and other retinoids [76]. Preclinical animal studies proved the chemopreventive activity of 9cUAB30 in reduction of mammary cancers (Table 1) [76, 77]. A first study among humans recently demonstrated a favorable toxicity and pharmacokinetic profile of 9cUAB30, and a phase I dose-escalation study is currently in progress.

Our laboratory has previously shown that the rexinoid LG100268 can prevent ER-negative/HER2 positive mammary tumors in preclinical mouse models [78] and delay TNBC development [79]. Moreover, combination therapy using LG100268 and a synthetic triterpenoid, CDDO-methyl amide, synergistically suppressed ER-negative/HER2 positive mammary tumors (Table 1) [70]. Future preclinical and clinical studies are needed to determine the future of retinoids and rexinoids as cancer-preventive agents. Other approaches, for example combination treatments may be a promising new strategy to reduce ER-negative breast cancer incidence.

### Statins

Statins inhibit 3-hydroxy-3-methyl-glutaryl-coenzyme A (HMG-CoA) reductase, which reduces the intracellular biosynthesis of cholesterol by reversibly inhibiting the conversion of HMG-CoA to mevalonate (Fig. 1) [80]. These lipid-lowering drugs (for example atorvastatin, cerivastatin, fluvastatin, lovastatin, simvastatin, and pravastatin) are commonly used to treat hypercholesterolemia, and thereby reduce the risk of cardiovascular diseases. Preclinical, clinical, and epidemiologic studies provide a rationale for evaluating lipophilic statins for breast cancer prevention [80–83]. Results from these studies are conflicting, however, resulting in inconsistency in the relationship between statin use and reduced incidence of breast cancer.

A decrease in risk of many types of cancer, including breast cancer, among statin users was observed in many studies [81, 84–88]. Conversely, two meta-analyses concluded that statin use and long-term statin use did not significantly affect breast cancer risk [89, 90]. A recently conducted study revealed no association of lipid-lowering drug use with reduced risk of breast cancer recurrence and breast cancer-specific mortality [91].

To investigate the biological effect of lipophilic statins in the prevention of breast cancer several biomarker modulation trials have been initiated [92, 93]. After short-term statin treatment of women with high-grade (DCIS or stage 1) breast cancer, proliferation was reduced and apoptosis increased (Table 2) [93]. However, another biomarker modulation trial of lovastatin among women with increased risk of breast cancer did not show any significant breast duct cytology changes after lovastatin therapy [94].

In the future, it will be necessary to conduct clinical trials among high-risk populations, in particular women at high risk of TNBC, to determine whether statins will be useful as preventive therapy.

### PARP Inhibitors

Poly(ADP-ribose) polymerases, particularly PARP1, are multifunctional enzymes best known for their repair of breaks in single-strand DNA. Inhibition of PARP-1 is a promising approach for targeted prevention of breast cancer, especially among women with deleterious BRCA mutations [95]. Several PARP inhibitors, for example iniparib (BSI-201), olaparib (AZD2281), rucaparib (AG014699), veliparib (ABT-888), and BMN-673, are currently in clinical development as cancer therapeutics for breast and ovarian cancer [95]. However, limited data exists on studies testing the efficacy of PARP inhibitors as a prevention agent. A recent preclinical study by Liby and Sporn demonstrated that olaparib (200 mg kg^−1^ diet) or veliparib (100 mg kg^−1^ diet) significantly delayed tumor development in BRCA1-deficient mice (Table 1) [96]. PARP inhibitors have not yet been tested for chemoprevention in clinical or biomarker modulation trials. However, PARP inhibitors may be found to be useful for cancer prevention in the future, considering approximately 55 to 65 % of BRCA1 mutation carriers will develop breast cancer by age 70 years [97].

### Other Promising Agents

Novel targeted drugs with demonstrated efficacy for treatment of breast cancer may also be useful for prevention of breast cancer. IGF-pathway inhibitors, for example cixutumumab or figitumumab, have been effective in the treatment of both ER-positive and ER-negative breast cancer [98]. However, little progress has been made in determining the usefulness of these drugs as preventive therapy, partly because of their known toxicity, for example hyperglycemia. PI3K/AKT/mTOR signaling is critical for regulation of cell growth and cell survival and is thus important in tumorigenesis [99]. mTOR, a serine-threonine kinase, acts as a downstream effector of the PI3K/AKT signaling pathway and phosphorylates multiple downstream kinases [100]. mTOR inhibitors (for example rapamycin, everolimus, sirolimus, and temsirolimus) may be useful as cancer-prevention agents. Rapamycin has been used in several preclinical studies (Table 1) which demonstrated that this drug is able to reduce tumor growth in ER-negative breast tumor mouse models [101, 102]. These initial positive results are encouraging for further development of mTOR inhibitors as cancer preventive agents.

A variety of triterpenoids have chemopreventive potential in breast cancer. Natural triterpenoids are abundantly found in marine sources, for example marine sponges, sea cucumbers, or marine algae [103], and have antiproliferative, antiangiogenic, anti-inflammatory, and pro-apoptotic activity [104]. CDDO esters and CDDO-Me have been shown to delay ER-negative mammary tumor formation in animal studies (Table 1) [70, 105].

### Natural Compounds

Although there is a strong interest in using more than one-hundred natural compounds [106] for cancer prevention, none of these dietary agents has been shown to consistently prevent breast cancer. Some of the most promising compounds include catechins (e.g., epigallocatechin gallate (EGCG), green tea extract) [107], curcumin [108], luteolin [109], carotenoids [110], omega-3-fatty acids [111], resveratrol [112–114], soy isoflavones [115, 116], and vitamin D [117, 118]. For example, green tea intake has been associated with reduced incidence of breast cancer, and a recent phase IB dose-escalation trial using 400–800 mg EGCG among women with a history of stage I to III hormone receptor-negative breast cancer demonstrated that this natural compound is well tolerated [107] (Table 2). On the basis of this positive result, a phase II trial testing the cancer-preventive effect of EGCG over a one year treatment period is currently in progress among postmenopausal women with high breast density. Many vitamins, in particular Vitamin D, are currently being tested as preventive agents among women at high risk of breast cancer. The VITAL trial (vitamin D and omega-3 fatty acids) is testing the daily intake of vitamin D_3_ (2000 IU), omega-3 fatty acids (1000 mg), the combination, or placebo among 20,000 healthy men and women for five years [119] (Table 2). The primary outcomes are cancer incidence, cardiovascular disease, stroke, and diabetes. Although natural products are a promising alternative cancer-prevention strategy, their potential efficacy in the prevention of ER-negative and, particularly, triple-negative breast cancer will be determined in the near future.

### How to Select Individuals for Preventive Therapy (Risk Stratification)

Accurate assessment of a women’s breast cancer risk on the basis of known risk factors is needed to decide who might benefit most from targeted preventive therapy. In particular, targeted chemoprevention will be used for women at high risk. For example, women with HER2-positive DCIS would benefit from anti-HER2 therapy after consideration of risk versus benefit factors. To this end, statistical models have been developed to help predict breast cancer risk on the basis of known risk factors. The most frequently used is the Gail model (reviewed elsewhere [120]), although this model is not able to specify a risk for solely ER-positive or solely ER-negative breast cancer. Each of the currently available risk-prediction models has its limitations and cannot be appropriately applied to all patients. Incorporation of known biomarkers, gene-expression profiles and pharmacogenetics, susceptibility genes, and breast density is needed to accurately identify women at particularly high risk of ER-negative breast cancer.

## Conclusions and Future Perspective

Because breast cancer comprises distinct subtypes, identification and development of effective and safe preventive therapy remains challenging. To successfully implement preventive therapy for all types of breast cancer, chemoprevention must change to personalized therapy. For example, several prevention trials among women with HER2-positive DCIS have demonstrated the potential of HER2-targeted drugs as chemopreventive agents for this subtype of breast cancer. The heterogeneous nature of TNBCs, which have multiple signaling pathways activated, necessitates multi-targeted approaches to effective TNBC prevention. Moreover, successful cancer prevention requires accurate identification of individuals at high risk of specific breast cancer subtypes. Such high-risk individuals are most likely to benefit from targeted preventive therapy.

Although many women qualify for preventive therapy, most high-risk women are not interested in using drugs for cancer prevention. This is partly because of the perception that the benefits do not outweigh the side effects. Whereas many women with cancer will tolerate side effects during their therapy, most women do not accept the same side effects of preventive therapy when healthy. To overcome this challenge, education of the public and medical community with evidence-based risk and benefit information is needed. Moreover, most preventive therapy is administered chronically for many years; other strategies, for example intermittent dosing schedules should be investigated to reduce common and rare serious side effects.

The success of breast cancer prevention research depends on molecularly targeted approaches, particularly for TNCB prevention, and the development of less toxic drugs that interrupt drivers of tumorigenesis.
